# The Transcription Factor Egr3 Is a Putative Component of the Microtubule Organizing Center in Mouse Oocytes

**DOI:** 10.1371/journal.pone.0094708

**Published:** 2014-04-10

**Authors:** Hyejin Shin, Sojung Kwon, Haengseok Song, Hyunjung Jade Lim

**Affiliations:** 1 Department of Biomedical Science & Technology, Institute of Biomedical Science & Technology, Konkuk University, Seoul, Korea; 2 Department of Biomedical Science, College of Life Science, CHA University, Seoul, Korea; China Agricultural University, China

## Abstract

The early growth response (Egr) family of zinc finger transcription factors consists of 4 members. During an investigation of Egr factor localization in mouse ovaries, we noted that Egr3 exhibits a subcellular localization that overlaps with the meiotic spindle in oocytes. Using Egr3-specific antibodies, we establish that Egr3 co-localizes with the spindle and cytosolic microtubule organizing centers (MTOCs) in oocytes during meiotic maturation. Notably, the Egr3 protein appears to accumulate around γ-tubulin in MTOCs. Nocodazole treatment, which induces microtubule depolymerization, resulted in the disruption of spindle formation and Egr3 localization, suggesting that Egr3 localization is dependent on the correct configuration of the spindle. Shortly after warming of vitrified oocytes, growing arrays of microtubules were observed near large clusters of Egr3. An in vitro microtubule interaction assay showed that Egr3 does not directly interact with polymerized microtubules. Egr3 localization on the spindle was sustained in early preimplantation mouse embryos, but this pattern did not persist until the blastocyst stage. Collectively, our result shows for the first time that the Egr3 a transcription factor may play a novel non-transcriptional function during microtubule organization in mouse oocytes.

## Introduction

The early growth response (Egr) family of zinc finger-containing transcription factors includes 4 members (Egr1–4) that participate in multiple physiological processes [1]–[6]. Egr1 plays a well-established role in regulating the transcription of the luteinizing hormone β subunit gene [7] and hormone responsiveness in the ovary [8]. A series of studies employing Egr3 deficient mice have shown that Egr3 is crucial for muscle spindle formation, dendrite morphogenesis, and target tissue innervation by sympathetic neurons [3], [9]. Egr3 is also known to be an estrogen-responsive gene that is involved in the estrogen signaling pathway in MCF7 human breast cancer cells [10]. However, the expression pattern and function of Egr3 in other estrogen-responsive organs has not been described. Egr4 deficient male mice are reportedly infertile, implicating Egr4 in male reproduction [11]. Consistent with this role, Egr4 is associated with the maintenance of the spermatogonia stem cell (SSC) pool in the rat testis [12]. An Egr family of protein in C. elegans, encoded by the early growth response homolog (egrh-1), plays a role in oocyte maturation and ovulation in the absence of sperm [13].

Mammalian oocytes are acentrosomal, meaning that there is no centrosome for spindle assembly [14], [15]. Instead, microtubule organizing centers (MTOCs) assemble spindles in these cells [14]. MTOCs drive the nucleation of microtubules, and the well-described pericentriolar proteins, γ-tubulin and pericentrin, are localized to these structures [16], [17]. During prophase, MTOCs are made de novo near the nucleus [18]. When the nuclear membrane disappears during germinal vesicle breakdown (GVBD), MTOCs leave from the periphery of the nucleus and are scattered in the ooplasm or accumulate near the spindle poles [19]. In mouse oocytes, a distinctive ring pattern of pericentrin forms at each spindle pole [17]. γ-tubulin production increases from prometaphase to metaphase I, and this protein precisely localizes to the meiotic spindle poles in maturing mouse oocytes [20]. The transition from the meiotic to mitotic spindle is gradual during early embryogenesis, with the meiotic spindle still visible in early preimplantation embryos [21]. Therefore, MTOCs in mammalian oocytes are packed with centrosomal proteins and serve the function of the microtubule assembly [18].

While investigating the expression of Egr factors in the mouse ovary, we observed a unique subcellular localization of Egr3 to the meiotic spindle of oocytes. This observation led us to investigate further the expression and distribution of Egr3 in mouse oocytes. Here, we show that the localization of Egr3 to the meiotic spindle depends on the presence of intact microtubules and its function may be closely associated with γ-tubulin-driven organization of MTOCs. Egr3 may play a unique non-transcriptional role in mouse oocytes.

## Results

### Egr3 localizes to the meiotic spindle of mouse oocytes within maturing follicles

**Figure 1:**
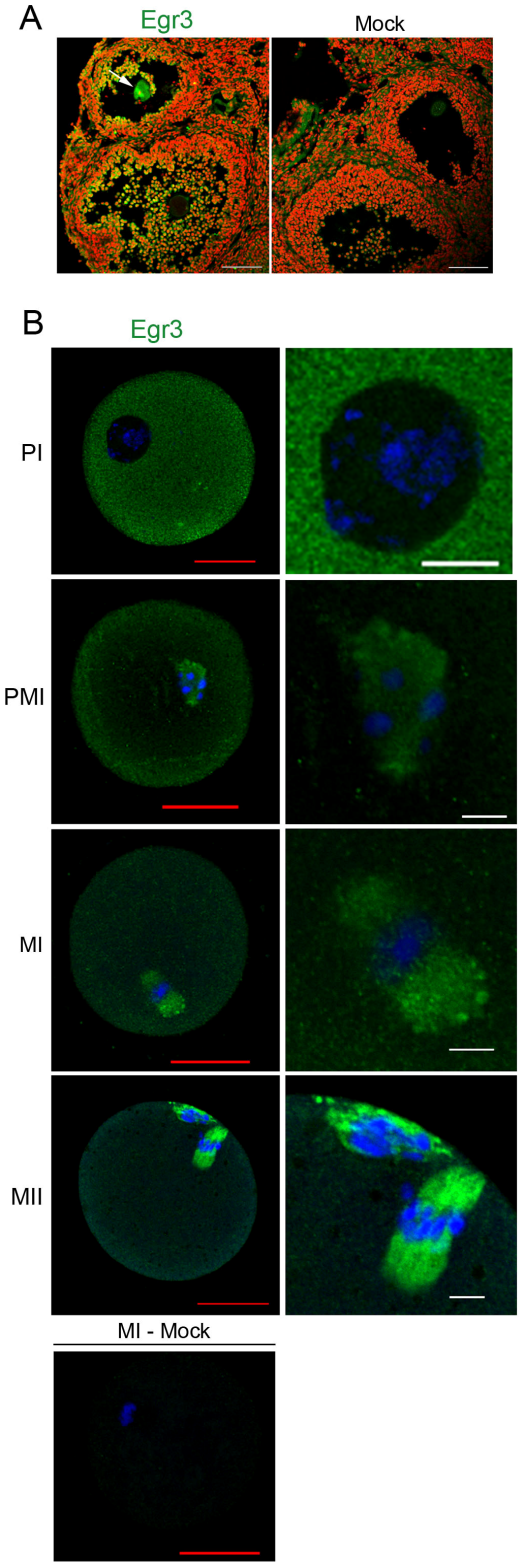
Subcellular localization of Egr3 protein in meiotic spindle of mouse oocytes. (A) Egr3 localization in a mouse oocyte within a growing follicle. Immunofluorescence staining of Egr3 was performed on ovarian cryosection fixed in acetone. The rabbit polyclonal anti-Egr3 antibody (Santa Cruz) was used at 2 μg/ml. Primary antibody was probed with goat anti-rabbit IgG-Alexa Fluor 488 antibody. The DNA was counter-stained with TO-PRO-3-iodide. White scale bar, 100 μm. Mock control, rabbit IgG. Green, Egr3; red, DNA. (B) Egr3 is localized to the meiotic spindle of mouse oocytes at all stages of maturation. Germinal vesicle (GV) stage oocytes were obtained by ovary puncture and were cultured in M16. Oocytes were fixed in 3.7% formaldehyde and were subjected to immunofluorescence staining with anti-Egr3 antibody (Santa Cruz). Oocytes in the left panel are shown at 60X and enlarged images of chromosome-containing areas are shown in the right panel. Mock control, rabbit IgG. PI, prophase I; PMI, prometaphase I (cultured for 3 h); MI, metaphase I (cultured for 8 h); MII, metaphase II (cultured for 12 h). Red scale bar, 30 μm; white scale bar, 10 μm. Green, Egr3; blue, DNA.

While investigating the expression of Egr transcription factors in the mouse ovary, we unexpectedly observed that Egr3 was localized to the meiotic spindles of maturing oocytes (Fig. 1A, arrow). We performed Egr3 immunofluorescence staining in parallel with Egr1, 2, and 4, and found that Egr3 was the only member of the Egr family that exhibited this subcellular localization in the mouse ovary (data not shown). Next, we confirmed the localization of Egr3 in isolated mouse oocytes at various stages meiotic maturation. Mouse oocytes were collected at 48 h post-PMSG (prophase I, PI) and were cultured in M16 media. Oocytes at PI, prometaphase I (PMI), metaphase I (MI), and metaphase II (MII) were subjected to Egr3 immunofluorescence staining. As shown in Fig. 1B, Egr3 protein was evenly distributed in the cytoplasm and in several puncta at the PI stage. Starting at PMI, Egr3 exhibited MTOC-like behavior. Accumulation of Egr3 was noted near condensing chromosomes and gradually exhibited spindle-like formation. This pattern was maintained up to MII. Two Egr3 antibodies from two different commercial sources showed this localization (data using one of them is shown in Fig. 1B). None of the other members of Egr transcription factors showed such pattern of localization (data not shown). Staining with anti-rabbit IgG did not produce any specific signal (Fig. 1B, bottom left).

### RT-PCR and Western blot analysis of Egr3 in mouse oocytes and somatic tissues

**Figure 2:**
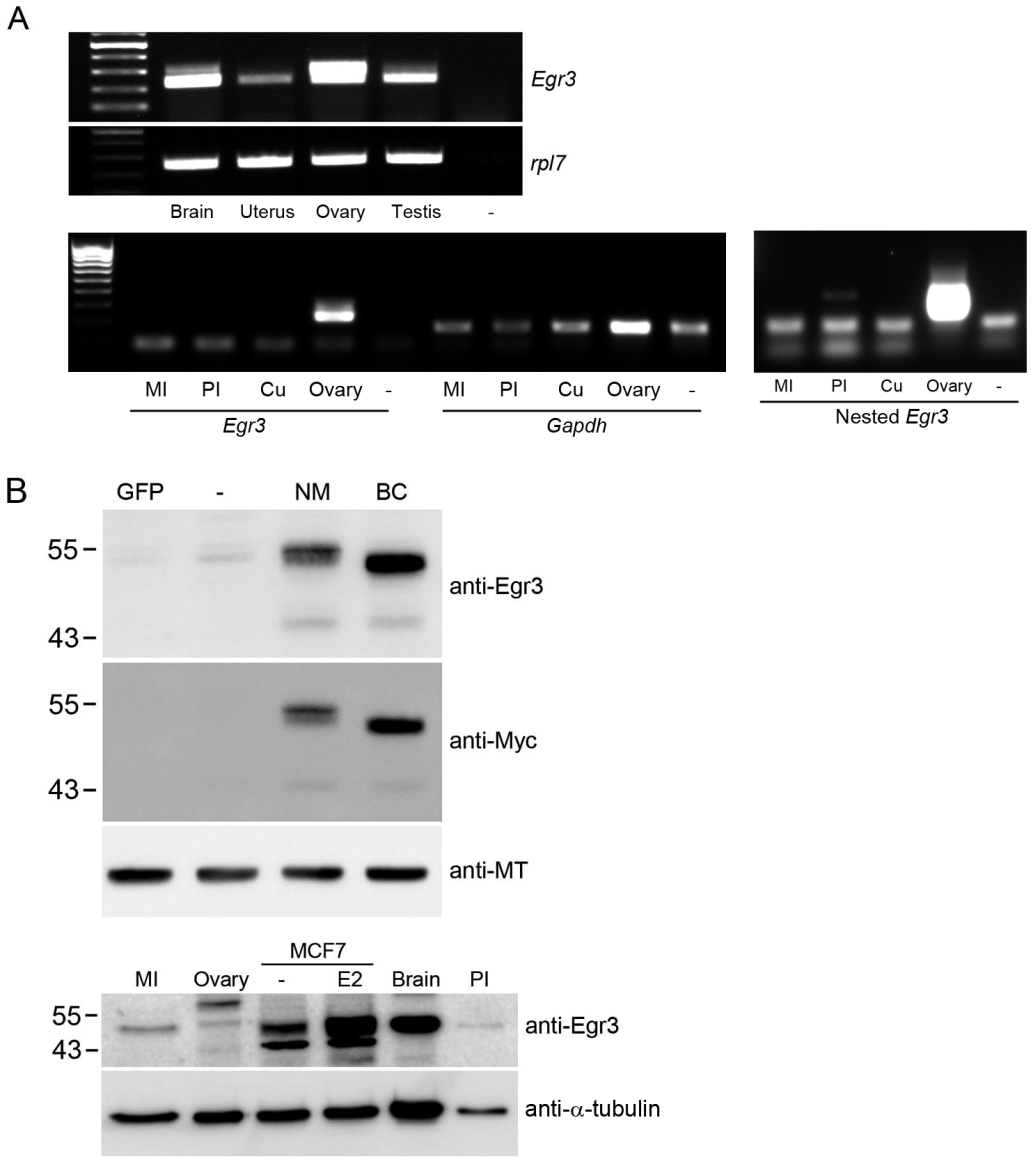
The expression of *Egr3* transcripts and proteins in mouse tissues and cultured cells. (A) An RT-PCR analysis of *Egr3* expression in various tissues. Expression was compared to that of ribosomal protein L7 (*rpl7*) or *Gapdh*. Oocytes at PI (130 oocytes) or MI stage (120 oocytes) were pooled and subjected to Egr3 RT-PCR and nested PCR. Cu, cumulus cells; -, no RT. (B) Western blot analysis of Egr3 in various tissue and cell types. Upper panel: 293 T cells transfected with CMV-GFP plasmid (GFP), empty plasmid (-), pcDNA3.1/myc-his-NM plasmid (NM), or pcDNA3.1/myc-his-BC plasmid (BC) were analyzed using Western blots probed with anti-Egr3 antibody (Santa Cruz). Fifty micrograms of each cell lysates were run on a 10% SDS-PAGE gel. Bottom panel: MI oocyte (268 GV oocytes cultured for 8 h in vitro), PI oocyte (112 oocytes), ovary, brain, and MCF7 cell (with or without estrogen treatment) lysates were analyzed by Western blotting. Two hundred micrograms of tissue extract was loaded in each lane of a 12% SDS-PAGE gel. The blots were stripped and tubulin was detected using an anti-α-tubulin antibody.

RT-PCR was performed to detect *Egr3* mRNA in mouse oocytes and other tissues. As shown in Fig. 2A, an *Egr3*-specific PCR product was obtained for brain, uterus, ovary, and testis, but not for PI and MI stage oocytes (Fig. 2A, lower panel, PI and MI). Nested PCR of PI oocyte cDNA generated a weak band (Fig. 2A), suggesting that the *Egr3* transcript was present at very low levels. Two *Egr3* RT-PCR products were observed in some tissues (Fig. 2A). By searching the GenBank database, we found two sequenced that are predicted to encode isoforms of mouse *Egr3* (NM_018781.2 and BC103568, herein abbreviated NM and BC, respectively). Thus, the two RT-PCR bands may indicate the expression of both transcripts in some tissues.

To rule out the possibility that the observed subcellular localization of Egr3 is an artifact, we tested the specificity of the Egr3 antibodies we used. We transfected 293 T cells with plasmids encoding each isoform of *Egr3* gene, and performed a Western blot on their cell lysates. As shown in Fig. 2B (upper panel), both the NM and BC isoforms are detected by the anti-Egr3 antibody. We further confirmed their specificity by using anti-Myc antibody, as the transfected plasmids possessed a Myc tag. Thus, these experiments demonstrate that the anti-Egr3 antibody is specific to both isoforms of Egr3 protein. Using this antibody, we performed a Western blot by using lysates from mouse oocytes and other tissues. Notably, PI and MI stage oocytes clearly show a single Egr3-specific band that is similar to the predominant band obtained for brain lysate. It has been shown that MCF7 cells respond to estrogen and express various genes including *Egr3* [10]. MCF7 cells express two isoforms of Egr3, and the level of the larger molecular weight isoform increases on estrogen treatment (Fig. 2B). This band corresponds to the one observed in mouse oocytes. One possibility is that mouse oocytes have Egr3 protein, but *Egr3* mRNA remains at a very low level as they undergo the maturation process. Alternatively, both *Egr3* transcripts are present, with the larger isoform (NM) being the predominant isoform translated in mouse oocytes and the other tissues we tested. With this information, we proceeded to perform more detailed analysis of the subcellular localization of Egr3 protein in mouse oocytes.

### Egr3 is associated with the spindle and MTOCs

**Figure 3:**
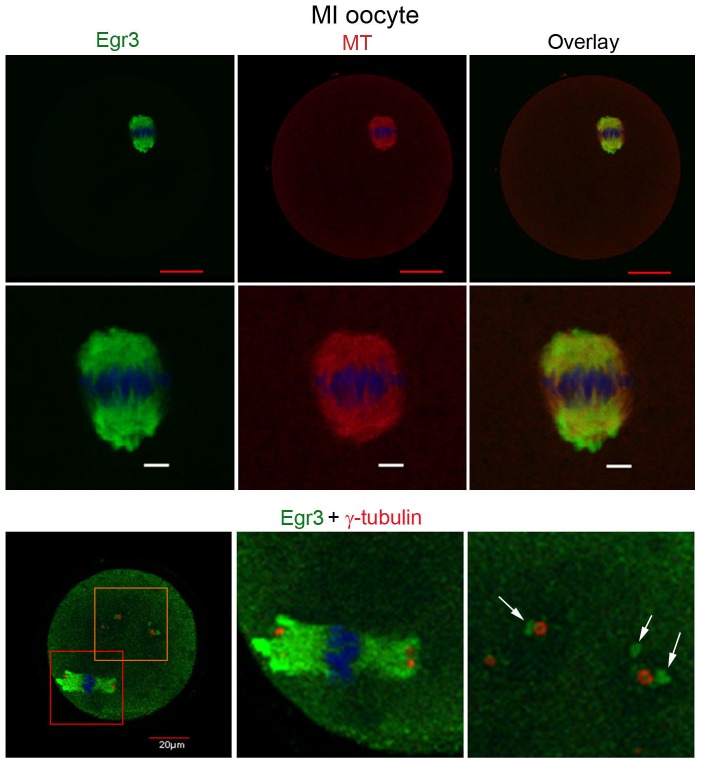
Immunofluorescence staining of Egr3 and tubulin proteins in MI oocytes. PI oocytes were cultured in vitro for 8% formaldehyde, and subjected to immunofluorescence staining with anti-Egr3 (Santa Cruz antibody in upper panel and Abcam antibody in low panel), anti-α-tubulin, or anti-γ-tubulin (Sigma-Aldrich) antibody. All the antibodies were used at 2 μg/ml. DNA was counter-stained with TO-PRO-3-iodide. Red scale bar, 30 μm; white scale bar, 5 μm. Green, Egr3; red, α-tubulin or γ-tubulin; blue, DNA. The arrows indicate the localization of Egr3 protein near γ-tubulin-positive MTOCs.

We co-stained MI stage oocytes with anti-α-tubulin and anti-Egr3 antibodies. As shown in Fig. 3, Egr3 is primarily localized to the spindle that is marked by α-tubulin staining in MI oocytes. While Egr3 staining mostly colocalized with the spindle, it did not completely overlap with spindle. Next, we co-stained oocytes with anti-γ-tubulin, a marker of MTOCs and spindle poles, as the staining pattern of Egr3 in oocytes ranging from PMI to MI was reminiscent to that of known MTOC components (Fig. 1B). γ-tubulin is localized to the spindle poles and cytoplasmic MTOCs (Fig. 3, lower panel) [19]. Notably, accumulations of patches of Egr3 were consistently observed near γ-tubulin (shown in red) in cytoplasmic MTOCs of MI oocytes (Fig. 3, arrows), suggesting that Egr3 is associated with the MTOCs in mouse oocytes.

### The localization of Egr3 is tightly associated with γ-tubulin organization

**Figure 4:**
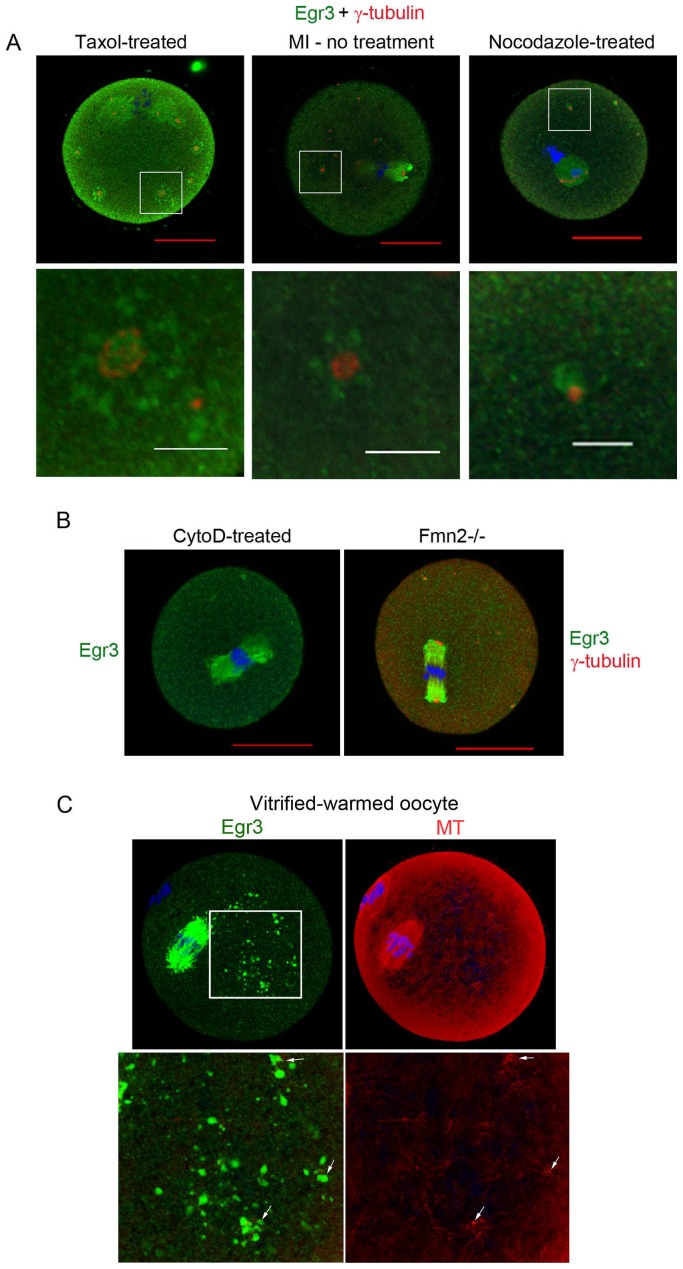
Egr3 localization is associated with microtubule organization in mouse oocytes. (A) MI oocytes were treated with taxol (microtubule stabilizer, 1 μM) or nocodazole (microtubule depolymerizer, 10 μg/ml) for 1–2 h and were subjected to immunofluorescence staining with anti-Egr3 antibody (Santa Cruz). Red scale bar, 30 μm; white scale bar, 10 μm. Green, Egr3; red, γ-tubulin; blue, DNA. (B) Localization of Egr3 is shown in MI oocyte treated with CytoD (actin depolymerizer, 10 μM) and in *Fmn2*-/- oocyte. *Fmn2*-/- oocyte was co-stained with anti-Egr3 (Santa Cruz) and anti-γ-tubulin antibodies. Red scale bar, 30 μm. Green, Egr3; red, γ-tubulin; blue, DNA. (C) The localization of Egr3 and microtubule at thawing of vitrified oocytes. Vitrified MII oocytes were stored in LN_2_ for 2 weeks. Oocytes were taken out from LN_2_, incubated in decreasing concentrations of sucrose, and then fixed immediately. These oocytes were subjected to immunofluorescence staining with anti-Egr3 (Abcam) and anti-α-tubulin antibodies. Arrows indicate the growing arrays of microtubules at the site of Egr3 accumulation. Green, Egr3; red, microtubule (MT).

To address if cytoskeletal organization in oocytes during meiosis regulates the subcellular localization of Egr3, we treated MI oocytes with drugs that modulate the polymerization of microtubules or actin filaments and co-stained them with Egr3 and γ-tubulin antibodies. Taxol and nocodazole were used to stabilize and depolymerize microtubule, respectively. Cytochalasin D (CytoD) was used to depolymerize actin filament in oocytes. As shown in Fig. 4A, taxol treatment increased the size of microtubule patches corresponding to the MTOCs, and the spindle appeared to be enlarged. Taxol treatment also increased the accumulation of Egr3 localized near γ-tubulin. Nocodazole treatment reduced the size of both spindle and MTOCs. As shown in Fig. 4A, the spindle had disintegrated in nocodazole-treated oocytes, and Egr3 no longer exhibited a spindle-like form. Egr3 localized near the γ-tubulin was also reduced. Thus, the amount of Egr3 accumulated near MTOCs appeared to be proportional to γ-tubulin buildup. Under cytoD treatment, the meiotic spindle maintained its original size and Egr3 localization was not affected (Fig. 4B). Formin-2 (Fmn2) is an actin nucleator and plays an important role in the organization of actin cables during spindle migration in mouse oocytes [22]. Defects in actin cable formation in *Fmn2* deficient (*Fmn2*-/-) oocytes lead to defects in spindle migration and polar body extrusion [23]. In *Fmn2*-/- oocytes, Egr3 maintains its localization near the meiotic spindle (Fig. 4B) further supporting the notion that actin filaments are not associated with the specific subcellular localization of Egr3 in mouse oocytes.

We next examined the relationship between Egr3 accumulation and microtubules during the recovery of oocytes from cryopreservation. MII stage oocytes were vitrified and kept in liquid nitrogen (LN_2_) for 2 weeks [24]. Oocytes were removed from the LN_2_, treated with decreasing concentrations of sucrose. Oocytes were then fixed and processed for Egr3 immunofluorescence staining. This process takes approximately 10 min at room temperature. In vitrified-warmed oocytes, growing arrays of microtubules were observed in numerous locations in the cytoplasm (Fig. 4C, white arrows). Accumulations of Egr3 staining (green) were consistently observed in proximity to the spots of cytoplasmic microtubules. Thus, the localization of Egr3 protein localization appears to be dynamically associated with the state of the oocyte microtubules.

### Egr3 co-localizes with the meiotic spindle, but does not directly interact with microtubules

**Figure 5:**
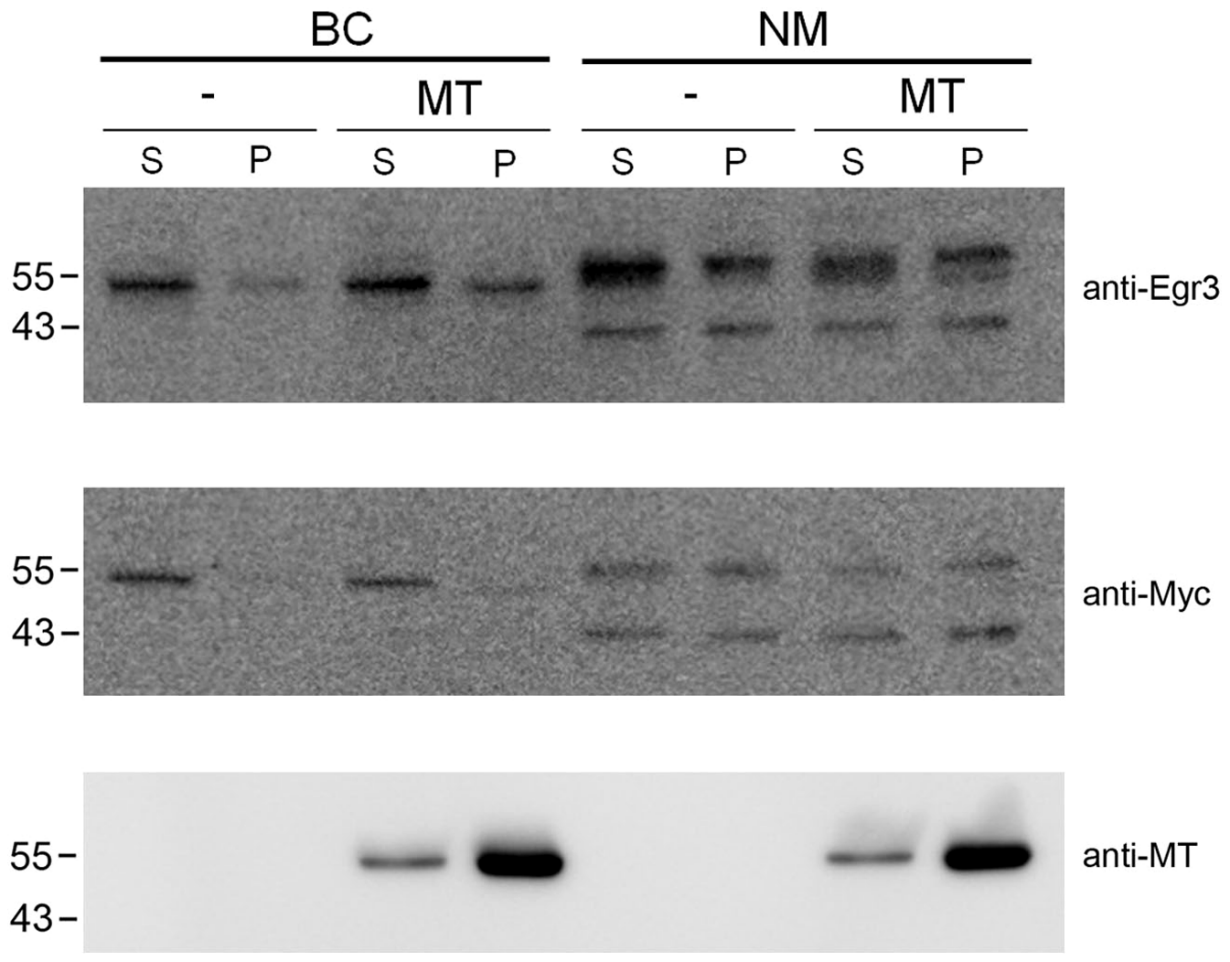
Microtubule interaction assay using in vitro translated Egr3 isoforms. To examine whether polymerized microtubules interact directly with Egr3 protein, we performed a microtubule-interaction assay. Tubulin from the bovine brain (5 μg/μl) was polymerized at 37°C and diluted to 0.2 μg/μl. Four microliters of in vitro translated lysates (NM or BC) and 50 μl of diluted MTs were incubated at room temperature for 30 min. The reaction was centrifuged at 100,000×g for 30 min to pellet the polymerized MTs. The supernatant (S) and pellet (P) were run on 10% SDS-PAGE and Western blotting was performed with anti-Egr3 or anti-Myc antibody which detects the epitope in the pcDNA3.1/myc-his vector. The antibodies were used at 1∶5000. The blots were stripped and tubulins were detected with anti-tubulin antibody. The data show that the presence of microtubules does not affect the quantity of Egr3 products in the supernatant and pellet, suggesting that there is no direct interaction between microtubules and Egr3.

The aforementioned results showing that Egr3 localization is regulated by microtubule dynamics led us to investigate whether Egr3 directly interacts with polymerized microtubules. We previously performed an in vitro assay to address if certain domain(s) in Fmn2 protein directly interacts with microtubules [25]. We adapted this assay to examine if in vitro translated Egr3 protein is pulled down when polymerized microtubules are spun down by ultracentrifugation. As shown in Fig. 5, the relative quantity of in vitro translated BC and NM isoforms are not affected by the presence of polymerized microtubules during ultracentrifugation. This result suggests that Egr3 protein does not co-precipitate with polymerized microtubules. Thus, Egr3 localization to the spindle requires the presence of an intact spindle in oocytes, but did not directly interact with microtubules in our in vitro assay.

### Egr3 localization in MCF7 cells, testis, and brain

**Figure 6:**
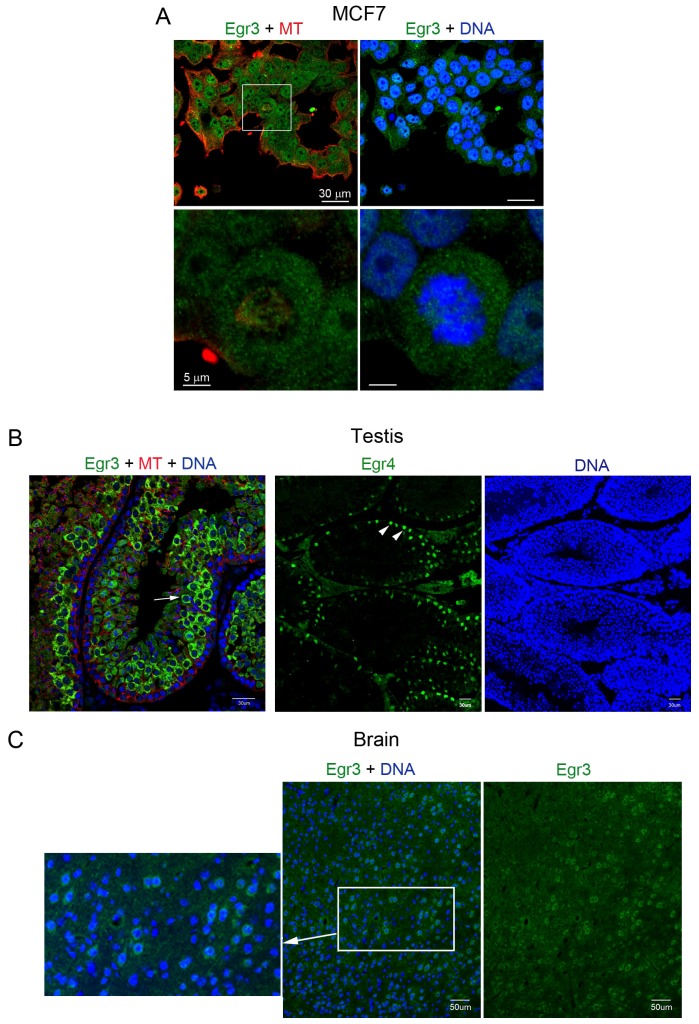
Egr3 localization in MCF7 cells and mouse testes. (A) Immunofluorescence staining of Egr3 in MCF7 human breast cancer cells. The cells were fixed in methanol and were stained with anti-Egr3 (Santa Cruz) and anti-α-tubulin (Sigma) antibodies. All the antibodies were used at 2 μg/ml. The white boxed area is shown enlarged in the lower panel. Green, Egr3; red, α-tubulin; blue, DNA. (B) Cryosections of 4-week-old mouse testis were fixed in 4% paraformaldehyde and subjected to immunofluorescence staining with anti-Egr3 (Santa Cruz) and anti-α-tubulin antibodies. Green, Egr3; red, α-tubulin; blue, DNA. (C) Cryosections of mouse brain were subjected to Egr3 immunofluorescence staining. The area within white rectangle is shown enlarged (left).

The subcellular localization of Egr3 protein in mouse oocytes is unique and atypical for a transcription factor. Next, we examined if this unique pattern is observed in other cell types. MCF7 human breast carcinoma cells have been shown to express *Egr3* [10]. We performed immunofluorescence staining of Egr3 in these cells, and as shown in Fig. 6A, Egr3 shows a diffused localization in both the nucleus and cytoplasm. In a dividing cell (white box, enlarged image is shown in the lower panel), Egr3 distribution (green) does not overlap with α-tubulin (red). In the mouse testis, Egr3 is strongly expressed in spermatocytes (Fig. 6B, white arrow). Egr4, whose mRNA expression in the rat testis was previously described, is predominantly localized to spermatogonial stem cells (Fig. 6B, white arrowheads). Thus, the expression patterns of Egr3 and Egr4 are mutually exclusive. Notably, Egr3 is primarily cytoplasmic in spermatocytes, whereas Egr4 localization is strictly nuclear. In the mouse brain, *Egr3* mRNA is reportedly present in several regions including cortex, hippocampus, and amygdala [26]. In the brain cortex, some neurons show an Egr3-positive signal in either the nucleus or the cytoplasm (Fig. 6C, enlarged image).

### Localization of Egr3 in preimplantation mouse embryos

**Figure 7:**
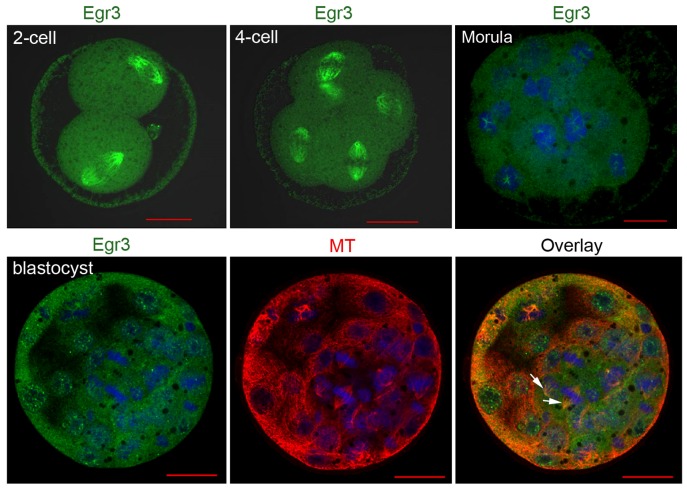
Immunofluorescence staining of Egr3 in preimplantation mouse embryos. Embryos at different developmental stages were fixed in 4% paraformaldehyde and subjected to immunofluorescence staining with anti-Egr3 (Santa Cruz) and anti-α-tubulin antibodies. The arrows indicate mitotic spindles in the blastomeres. Green, Egr3; red, α-tubulin; blue, DNA.

As previously mentioned, the meiotic spindles of oocytes are still present in early preimplantation embryos and gradually shifted to mitotic spindle as development proceeds [21]. Thus, we examined if Egr3 localization changes during the preimplantation embryonic development. We examined the localization of Egr3 in preimplantation embryos at 2-cell, 4-cell, morula, and blastocyst stages by performed immunofluorescence staining. In 2- and 4-cell stage embryos, Egr3 continues to exhibit a spindle-like localization (Fig. 7). In morula stage embryo shown in Fig. 7, blastomeres are at PMI of mitosis, and Egr3 accumulation was observed near condensing chromosomes. In contrast, nuclear localization was prominent in several blastomeres of the blastocyst stage embryos, and the spindle-like localization was not visible in dividing blastomeres (Fig. 7, arrows). Thus, the spindle-like localization of Egr3 seems to be restricted to maturating oocytes and early preimplantation embryos in mice.

## Discussion

The roles of Egr3 in muscle spindle morphogenesis and neurological functions have been established in a series of studies employing *Egr3* deficient mice [3], [9], [27], [28]. These studies showed that Egr3 plays important roles in the formation of muscle spindles, learning and memory, and target tissue innervation and dendrite morphogenesis in sympathetic neurons [3], [4], [9], [28]. While closely related family members, Egr1 and 4, are shown to mediate specific reproductive functions, expression and function of Egr3 in reproductive systems has not been explored. A key finding in this study is that Egr3 protein localizes near the meiotic spindle in maturating mouse oocytes, and this pattern correlates with the accumulation of γ-tubulin (Fig. 1 and 3). The amount of Egr3 accumulation in the ooplasm depends on γ-tubulin accumulation nearby (Fig. 4), suggesting that Egr3 and γ-tubulin are possibly functionally connected. Thus, Egr3 appears to be a novel component of the MTOCs, but does not directly interact with γ-tubulin. The main components of oocyte MTOCs, γ-tubulin and pericentrin, interact with each other [17], [29], [30]. We recently identified mouse diaphanous 2 (mDia2) as a new component of MTOCs and spindle pole, co-localizing with γ-tubulin and pericentrin [24]. Egr3 is unique in that it maintains distance from clusters of γ-tubulin, yet its localization appears to be associated with the organization and amount of γ-tubulin nearby (Fig. 4). This subcellular localization alludes to the possibility that Egr3 possesses a non-transcriptional function in the modulation of microtubule growth.

While several studies implicate Egr3 in transcriptional regulation, information regarding its subcellular localization is limited [31]. A study showed that Egr3 exhibits nuclear localization in a prostate cancer cell line [31]. The unique pattern that we observe in mouse oocytes has never been reported. For Egr1, it was revealed in the same study that it localizes to the mitotic spindle and microtubule meshwork in prostate cancer cell lines [31]. Because this finding has not been examined in other cellular systems, it is unclear if Egr transcription factors have an uncharacterized function in cytoskeleton organization. It has been shown that Kaiso, a transcriptional repressor containing zinc finger domains for DNA binding domains, localizes to the mitotic spindle and is a potential constituent of pericentriolar material [32]. This is another example of a transcriptional regulator with a potential role in cytoskeleton organization. Two isoforms of Egr3 with differential DNA binding abilities have been previously reported in rats [33]. The mouse *Egr3* gene also encodes two isoforms with differing N-terminal sequences. The larger isoform (NM), appears to be the most prevalent form in brain tissue and oocytes (Fig. 2B). Presently, it is not clear if the availability of specific isoforms is associated with the non-nuclear localization of Egr3 protein.

To the best of our knowledge, this is the first report stating that Egr3 exhibits a strong cytoplasmic localization in spermatocytes (Fig. 6). In microarray experiments using self-renewing spermatogonial stem cells (SSCs) isolated from young mice, *Egr3* and *Egr2* were identified as genes that respond to the withdrawal and replacement of growth factors essential for self-renewal and survival of SSCs in vitro [34]. These observations conflict with our data showing that Egr3 strongly localizes to spermatocytes, but not SSCs. It would be interesting to address this discrepancy in future work.

While our work suggests a new role for Egr3, it remains descriptive and warrants further investigation using gene-targeted mouse models. Because *Egr3* deficient mice show perinatal mortality with severe neurological defects [3], this model would not yield useful information regarding the role of Egr3 in oocyte biology. Recently, a floxed *Egr3* mouse line was generated [9]. Using this mouse line, a phenotypic analysis after oocyte-specific deletion of *Egr3* would be possible. Phenotypic analyses of *Egr3* deficient mice have been centered on the various neuronal deficits these mice display [9].Whether a non-transcriptional mechanism of Egr3 action is involved in its role in neuronal function remains unclear. To the best of our knowledge, this work provides the first evidence that Egr3 may be associated with microtubule organization in mouse oocytes; however, further work is required to elucidate the mechanism of its action in oocytes and other cells.

## Materials and Methods

### Ethics statement

Mice were maintained in accordance with the policies of the Konkuk University Institutional Animal Care and Use Committee. The study conducted herein was approved by the Konkuk University IACUC (approval number KU12081).

### Mice and tissue preparation

Mice were cared for in a controlled barrier facility within the College of Veterinary Medicine, Konkuk University. The *Fmn2* deficient mouse line was a generous gift from Drs. B. Leader and P. Leder (Harvard Medical School, Boston, MA, USA) [22]. To obtain tissues for RT-PCR and Western blotting, ICR female and male mice (n = 2 each) were sacrificed by cervical dislocation. Brain, ovary, and testis were dissected from them and placed in TRI Reagent (Molecular Research Center, Cincinnati, OH, USA) for RNA preparation, cryosectioning, and Western blotting.

### Culture of mouse oocytes and embryos

Four week-old ICR mice were sacrificed by cervical dislocation 48 h after an injection of pregnant mare's serum gonadotropin (PMSG, 5IU/mouse). Cumulus oocyte complexes (COCs) were collected by puncturing the ovaries in M2 media (Sigma-Aldrich, St. Louis, MO, USA). Each mouse generally ovulates about 17–20 COCs. Cumulus cells were mechanically removed from the oocytes by a mouth-controlled pipette. Collected oocytes were cultured in M16 media (Sigma-Aldrich) for 3 h (prometaphase I, PMI), 8 h (metaphase I, MI), or 12–16 h (metaphase II, polar body) in 37°C, 5% CO_2_ incubator in a drop of M16 media covered with mineral oil [25]. In some experiments, the oocytes were treated with nocodazole (10 μg/ml), taxol (1 μM) or cytochalasin D (cytoD, 10 μM, Sigma-Aldrich) by adding it to the culture media. Naturally mated female mice were sacrificed on day 2, and oviducts were flushed to obtain 2-cell stage embryos. Uteri from day 4 pregnant mice were flushed to obtain blastocysts. Embryos were cultured in KSOM-AA (Millipore, Billerica, MA, USA). Embryos were treated with nocodazole (3 μg/ml) for 12 h (Schuh and Ellenberg, 2007) and recovered in media for 40 min before they were processed for immunofluorescence staining.

### Vitrification and warming of mouse oocytes

As previously described, the vitrification solutions contained ethylene glycol (EG, Sigma-Aldrich) and dimethyl sulfoxide (DMSO, Sigma-Aldrich) as cryoprotectants [35]. The oocytes were pre-equilibrated in equilibration solution (7.5% EG, 7.5% DMSO, and 0.5 M sucrose; Fisher Scientific, St. Louis, MO, USA) for 2.5 min at room temperature [35]. The oocytes were then transferred to the vitrification solution (15% EG, 15% DMSO, and 0.5 M sucrose). After 20 s of incubation, the oocytes were loaded onto Cryotop strips (Kitazato Corporation, Shizuoka, Japan) and kept in LN_2_ for 2 weeks [36]. For warming, the Cryotop strip was taken out of the LN_2_ and were directly placed in the thawing media (0.5 M sucrose and 20% FBS in PBS) for 2.5 min. The thawed oocytes were collected and sequentially incubated in solutions containing decreasing concentrations of sucrose (0.25 M, 0.125 M, and 0 M) for 2.5 min each. Oocytes were then fixed to observe growing microtubules from MTOCs [24].

### RT-PCR

Total RNA was extracted using TRI Reagent according to the manufacturer's protocol. For each tissue, 2 μg of total RNA was reverse transcribed as described previously, by using M-MLV reverse transcriptase (Beams Biotechnology, Sungnam, Korea) [25]. Total RNA was extracted from oocytes at prophase I (PI, 130 oocytes) or metaphase I (MI, 120 oocytes) stage. Cumulus cells from COCs were also pooled (130 COCs) and RNA was extracted. Generally, about 3 μg of cDNA can be obtained from 100 oocytes. For PCR reactions using oocyte cDNA, we used ∼1.4 μg cDNA. PCR was performed using the Prime Taq Premix (Genet Bio, Daejeon, Korea). The following primers were used: 5′-CAA TCT GTA CCC CGA GGA GAT-3′ and 5′-AGG AGA GTC GAA AGC GAA CTT-3′ for *Egr3* (product size  = 217 bp); 5′-TCA ATG GAG TAA GCC CAA AG-3′ and 5′-CAA GAG ACC GAG CAA TCA AG-3′ for *rpl7* (product size  = 246 bp); 5′-TGC CCC CAT GTT TGT GAT G-3′ and 5′-TGT GGT CAT GAG CCC TTC C-3′ for *Gapdh* (product size  = 151 bp). For the nested PCR of *Egr3* in mouse oocytes, a pair of internal primers was designed as follows: 5′-AGC AGC GAC TCG GTA GCC CA-3′ and 5′-CAC GGT CTT GTT GCC GGG GG-3′ (product size  = 135 bp). Annealing temperature is 59°C for all reactions. For PCR reactions using tissue and oocyte cDNAs as template were run for 33 and 45 cycles, respectively.

### Plasmid construction and in vitro translation

The full-length open reading frame of mouse *Egr3* was PCR amplified from cDNA that was generated from total RNA from the mouse ovary (NM_018781.2) or purchased from DNAFORM (Kanagawa, Japan; BC103568, I.M.A.G.E clone ID: 40045218). Both forms of cDNA were cloned into pCDNA3.1-Myc-His vector (Invitrogen, Carlsbad, CA, USA). For microtubule interaction assay (see below), in vitro translation was performed using TNT T7 Coupled Reticulocyte Lysate System Kit (Promega, Madison, WI, USA) following the manufacturer's instruction. For microtubule interaction assay, two Egr3 constructs were produced. Full-length Egr3 (NM_018781.2) of 1164 bp (1–387 amino acids) and its isoform (BC103568) of 1050 bp (1–349 amino acids) were each cloned into pcDNA3.1/myc-His A vector (Invitrogen).

### Microtubule interaction assay

Tubulins (Cytoskeletons, Inc., Denver, CO, USA) were polymerized at 37°C and diluted to 0.2 mg/ml in dilution buffer (80 mM PIPES, pH 7.0, 1 mM MgCl_2_, 1 mM EGTA, 20 mM taxol and 0.05% NP-40) [25]. In vitro translated lysate (4 μl) and diluted microtubules (50 μl) were incubated at room temperature for 30 min. The reaction mixture was ultracentrifugated (100 000 g) to pellet polymerized microtubules. The pellet (polymerized microtubule) and supernatant were loaded onto a 10% SDS-polyacrylamide gel and subjected to Western blotting with anti-Express antibody [25]. Anti-Egr3 (Santa Cruz Biotechnology, CA, USA) and anti-c-Myc (Clontech Laboratories, Inc., CA, USA) were also used in some experiments.

### Protein extraction and Western blotting

Protein extraction and Western blotting were performed as described previously [25]. 293 T cell were transfected with Myc-tagged *Egr3* cDNAs described above. MCF7 human breast carcinoma cells were shown to express *Egr3* mRNA upon estrogen treatment [10]. These two cell lines were used as positive controls for Egr3 Western blotting. Cell lysate were prepared in RIPA buffer [50 mM Tris (pH 7.5), 150 mM NaCl, 1% NP-40, 0.5% Na-deoxycholate, 0.1% SDS, 1 mM DTT, 1 mM PMSF]. An aliquot of Complete protease inhibitor cocktail (Roche, Indianapolis, IN, USA) was added to the lysate. Mouse brain and ovary were homogenized with Polytron homogenizer (Brinkmann, Westbury, NY, USA) in RIPA buffer and centrifuged at 13000 rpm. The protein quantity was measured with BCA assays (Thermo Scientific, Rockford, IL, USA). Denuded oocytes were directly collected in 1X sample buffer [62.5 mM Tris-Cl (pH 6.8), 1.25% SDS, 25% Glycerol, 0.5% β-mercaptoethanol]. Fifty micrograms of protein were loaded per lane and separated in 10 or 12% SDS-PAGE gels. For oocyte samples, PI stage (112 oocytes) and MI stage (268 oocytes) were used. PVDF membrane was used for transfer. After blocking of membrane with 5% skim milk in TBS, the membrane was incubated with primary antibody for 2 h at room temperature. Anti-α+β-tubulin (Sigma-Aldrich) and Anti-Egr3 (Santa Cruz Biotechnology) are used for 1∶2000 and 1∶500, respectively. To detect exogenous Egr3 proteins, anti-Myc antibody (Clontech, Mountain View, CA, USA) were used at 1∶2000. HRP-conjugated goat anti-rabbit and goat anti-mouse antibodies (GeneDEPOT, Barker, TX, USA), were used at 1∶10,000. Super Signal West Femto ECL reagent (Thermo Scientific, Wilmington, DE, USA) was used. Chemiluminescence signal was detected by LAS3000 (FUJIFILM, Tokyo, Japan).

### Immunofluorescence staining

Immunofluorescence staining using the mouse ovarian and testis sections was performed as described previously [37]. MCF-7 cells were grown on coverslips inserted in 6-well plate. Nocodazole (100 ng/ml) was added to the media to block mitosis for 12 h. To stain microtubules, methanol was used as fixative. Immunofluorescence staining of the oocytes was performed using a drop culture system [24]. Oocytes were fixed in 3.7% formaldehyde in PBS for 10 min and permeabilized in PBS with 0.25% triton X-100 for 10 min. Embryos were fixed in 4% paraformaldehyde for 30 min and permeabilized in PBS with 0.1% triton X-100. Samples were incubated with 2 μg/ml antibodies of anti-Egr3 (Santa Cruz Biotechnology or Abcam), anti-Egr4 (Santa Cruz), anti-γ-tubulin (clone GTU 88, Sigma), or anti-α-tubulin [YL1/2] (Abcam) in 2% BSA in PBS for overnight at 4°C. Alexa Fluor 488-labeled goat anti-rabbit IgG (Invitrogen) were used as the secondary antibody to detect anti-Egr3 antibody. Alexa Fluor 568-labeled donkey anti-mouse IgG was used to detect anti-γ-tubulin antibody. For microtubule detection, Alexa Fluor 594-labeled chicken anti-rat IgG was used. Samples were counterstained with TO-PRO-3-iodide (Invitrogen) at 1∶500 in PBS for 20 min at room temperature in dark. After a final wash in 2% BSA/PBS, the oocytes were directly placed onto a glass slide and covered with a glass coverslip. Rabbit IgG was used at the same concentration as a mock control in each experiment.

### Confocal microscopy

Images were obtained using the Olympus Fluoview FV1000 Confocal Microscope, with lasers at 458, 488, 515, 543, and 633 nm wavelengths (Tokyo, Japan). Fluorescence intensities were quantified using the Fluoview version 1.5 software, a platform associated with the confocal microscope.
